# A novel and accurate deep learning-based Covid-19 diagnostic model for heart patients

**DOI:** 10.1007/s11760-023-02561-8

**Published:** 2023-05-19

**Authors:** Ahmed Hassan, Mohamed Elhoseny, Mohammed Kayed

**Affiliations:** 1 Faculty of Science, Beni-Suef University, Beni-Suef, 62511 Egypt; 2 Faculty of Computers and Information, Mansoura University, Mansoura, 35516 Egypt; 3 Faculty of Computers and Artificial Intelligence, Beni-Suef University, Beni-Suef, 62511 Egypt

**Keywords:** Coronavirus, Electro diagrams, Deep learning, Heart patients

## Abstract

Using radiographic changes of COVID-19 in the medical images, artificial intelligence techniques such as deep learning are used to extract some graphical features of COVID-19 and present a Covid-19 diagnostic tool. Differently from previous works that focus on using deep learning to analyze CT scans or X-ray images, this paper uses deep learning to scan electro diagram (ECG) images to diagnose Covid-19. Covid-19 patients with heart disease are the most people exposed to violent symptoms of Covid-19 and death. This shows that there is a special, unclear relation (until now) and parameters between covid-19 and heart disease. So, as previous works, using a general diagnostic model to detect covid-19 from all patients, based on the same rules, is not accurate as we prove later in the practical section of our paper because the model faces dispersion in the data during the training process. So, this paper aims to propose a novel model that focuses on diagnosing accurately Covid-19 for heart patients only to increase the accuracy and to reduce the waiting time of a heart patient to perform a covid-19 diagnosis. Also, we handle the only one existed dataset that contains ECGs of Covid-19 patients and produce a new version, with the help of a heart diseases expert, which consists of two classes: ECGs of heart patients with positive Covid-19 and ECGs of heart patients with negative Covid-19 cases. This dataset will help medical experts and data scientists to study the relation between Covid-19 and heart patients. We achieve overall accuracy, sensitivity and specificity 99.1%, 99% and 100%, respectively.

**Supplementary Information:**

The online version contains supplementary material available at 10.1007/s11760-023-02561-8.

## Introduction

Covid-19 is a health disaster wherein there are 43 million Covid-19 positive cases and 1.2 million people have died as a result. It is necessary to develop an automatic, early and accurate COVID-19 diagnostic mechanism. The disease is typically detected using reverse-transcription polymerase chain reaction (RT-PCR) testing. In spite of this, RT-PCR has been found that the sensitivity of it is not high enough for early detection of COVID-19. Also, the supply of kits of RT-PCR is different from a country to another and many developing countries, that have a big number of cases and deaths, are in short supply of RT-PCR.

Recently, deep learning technology has achieved a great success in the field of medical imaging due to its high feature extraction capability [1, 2]. Recent research shows that artificial intelligence techniques can surpass the human experts in medical image diagnosis tasks, including also the lung diseases. The AI diagnostic algorithms also have the advantages of high efficiency and easy deployment at large scale. Deep learning techniques have been successfully used in many medical problems such as skin cancer classification [3, 4], lung segmentation [5], brain disease detection [6], pneumonia diagnosis from chest X-ray images, breast cancer detection, and fundus image segmentation. AI techniques is helpful in getting rid of disadvantages such as the unavailability of significant number of RT-PCR test kits, and the much waiting time of check results. Also, there has been many publicly available medical images for healthy cases, also for patients suffering from various pandemics such as Covid-19. So, this enables the researchers to analyze the medical images using AI techniques and identify patterns that may result in automatic diagnosis of Covid-19.

Covid-19 patients with heart disease are the most people that exposed to violent symptoms of Covid-19 and death [7–9]. This shows that there is a special and unclear relation (until now) and parameters between covid-19 and heart disease. So, as previous works, using a general diagnostic model to diagnosis covid-19 from all patients based on the same rules, whether having heart disease or other chronic disease or not, is not accurate, as we prove later in the practical section of our paper. In all areas of cardiac care, the sooner an accurate diagnosis is made, the likelihood of a full recovery significantly increases. So, this paper aims to propose a model that focuses on diagnosing accurately Covid-19 for heart patients only to increase the accuracy and to reduce the waiting time of a heart patient to perform a covid-19 diagnosis because this diagnostic model is only for heart patient. This has a very important contribution in saving heart patients early from Covid-19-violent symptoms and death. Besides this, heart patients cannot wait for developing a general Covid-19 diagnostic model, that is suitable to all people, because of their dangerous case. So, in this paper, we have to study this point that developing a Covid19 diagnostic model for heart patient only that is also more accurate than the general diagnostic models, as we practically clear later.

Here, we can list the contributions of our proposed model as shown below:

1. As a novel approach in Covid-19 diagnosis, we are the first to present an accurate diagnostic model for Heart patients only, compared to previous works that present a general diagnostic model to any one that cause a dispersion in the training process and affects the performance of the model, as we prove practically in the practical section.
2. Differently from previous works that focus on using X-ray or CT-scan dataset for their Covid-19 diagnostic models, we use ECGs images which are recently proved that ECGs show some specific features caused by Covid-19 [10]. To the best of our knowledge there is only one dataset, that contains ECGs of Covid-19 patients, which was published recently [11].
3. We handle this dataset [11] to be suitable to our model with the help of a heart diseases expert. We produce a new version of the dataset that consists of two classes: ECGs of heart patients with positive Covid-19 cases and ECGs of heart patients with negative Covid-19 cases.

This paper is organized as follow:Sect. 2 discusses the existing literature in the field of COVID-19; proposed classification model is discussed in the Sect. 3.3; performance analyses are discussed in the Sect. 4; the Sect. 5 concludes the paper.

## Related works

**Fig. 1:**
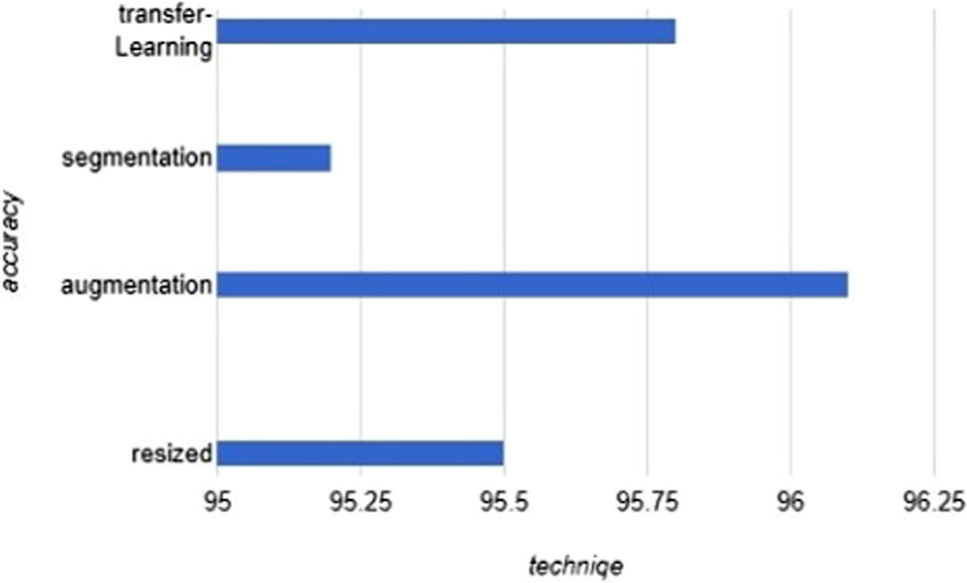
Novel practical comparison among pre-processing techniques for Covid-19 datasets

In this section, we firstly collect and summarize the active research tracks and open challenges of applying deep learning techniques to face Covid-19 pandemic, as shown in Table 1. Also in this section, we illustrate and compare the most high cited previous works that presented deep learning-based Covid-19 diagnostic model, as shown in Supplementary Table 2. We compare these works based on four basic dimensions: (1) the dataset (2) the pre-processing techniques to handle the dataset to increase the performance (3) the key points of the paper (4) the results. Covid-19 is a new pandemic, so the dataset of this pandemic stills limited and requires some pre-processing techniques to present the required performance. We compare these pre-processing techniques based on the average accuracy achieved by the most 10 high cited papers that use this technique to handle the dataset, as shown in Fig. 1.

**Table 1:** Summarization of the research tracks and open challenges of using deep learning in Covid19

Target	Research tracks	Research challenges
Diagnosis	CT scan-based diagnostic models [12] X rays-based diagnostic models [13, 14]	Unavailability of large datasets with also efficient-quality images for training Most DL models are trained for 2D images, however CT images are usually 3D Covid-19 stills unclear, so the same Covid19 case may be disagreed on it among medical experts. So, the labels of the dataset are not very accurate AI applications basically depend on big amounts of labeled data and less interaction with human experts. The basic challenge is how to decrease the labeling cost when time and human resources are limited
Covid-19 drugs	Drugs repurposing [15, 16] Drugs discovery [17]	Most works focus on drug-disease associations, but the relationships between drug–drug interactions must be considered in the model Most works depend in their predictions on general symptoms like fever, cough that may occur to any one due to climate change. This makes the predictions inaccurate
Prevention measures	Facial mask detection-based DL [18] Social-distancing alarm-based DL [19]	Most these DL-based models utilize image processing techniques, however in real crowded places or vehicles, where we want to apply these prevention measures, the faces of some people are hidden partially or completely behind the crowd. So, these applications do not achieve the required results in an efficient manner
Handling Covid19 side- effects	Fraud transactions during the pandemic-based deep learning [20] Predicting the most suitable educational system for the country during Covid-19. [21] Because of Covid-19, fuel demand decreases and the price of oil future becomes negative. So, in [22], a deep learning-based model is proposed that uses information such as travel activities and fuel usage to develop a model to predict the US gasoline demand in the medium-term and the effect of government interventions Predicting the people at a high risk of later chronic health disorders because of the big distress during the pandemic [23] Fake healthy information or news detection-based deep learning during Covid-19 [24]	Hard implementation and time consuming challenge due to the difference among language and accents in applications such as misinformation or fake news detections during Covid-19 based deep learning Applications such as predicting the suitable education system must include forecasting for the pandemic status at the coming period. Because the model may forecast based on the current status, but these status may change soon

### Discussion

 We found that, as shown in Supplementary Table 2, most papers that used X-ray images, as dataset for their models, achieved larger accuracy from the papers that used CT-scan images, as dataset for their models. So, the researchers were able to achieve efficient results, if they focused on using X-ray datasets.

Deep learning-based Covid-19 diagnostic models suffer from a common dangerous challenge that is the small number of Covid-19 samples existed in the public datasets. This may lead to poor results. Model [25] achieves the highest sensitivity and specificity compared to other models, in spite of it has very low Covid-19 samples in its corresponding dataset. It handles this challenge by using efficient technique which is data augmentation. Data Augmentation is a technique used to increase the size of specific class’s samples in an efficient manner. This solve the unbalance problem between covid-19’s samples and other class’s samples. We can also note that the papers such as [13, 14] that use efficient transfer learning have achieved very efficient results, wherein transfer learning is suitable to the training of small dataset such as Covid-19 datasets. So, the researchers have to focus on the techniques that handle the problem of Covid-19 small datasets such as efficient transfer learning and augmentation. In our point of view, results of paper [26] are not accurate because the used dataset consists of 1020 CT slices for only 186 patients (Covid and non-Covid). So, same patient has in average 5 CT slices. So, the CT-slice for the same case may repeat in the train and test set. This leads to raise the accuracy but not in an efficient way.

Recently, we have noted that some previous works trained their Covid-19 diagnostic model based on ECG samples, and these works achieved very reasonable accuracy [27–29]. Compared to all previous works, in this paper, we study the efficiency and accuracy level of developing Covid-19 diagnostic model for a certain category of patients, such as heart patients, exploiting the hidden and probable links between Covid-19 and heart diseases to achieve a more accurate diagnostic model. Besides this, heart patients cannot wait for developing a general Covid-19 diagnostic model, that is suitable to all people, because of their dangerous cases. So, in this paper, we have to study this point that developing a Covid19 diagnostic model for heart patients only that is also more accurate than the general diagnostic models, as we practically clear later.

## Methodology

In this section, information will be presented about the used COVID-19 dataset in details, the architectures used for classification which is based on combining transfer learning and ensemble learning. The steps of our proposed model are given.

### Dataset generation

Besides this, a team of researchers from New York University “Langone Medical Center”, USA, has found that the clinical severity of patients with COVID-19 disease can be predicted by analyzing the value of troponin elevation and electrocardiographic (ECG) abnormalities [10]. So, our model, based on an ECG dataset, predicts whether a heart patient is positive or negative Covid-19 case. This study [11] includes the dataset of ECG images of Cardiac and COVID-19 patients. This dataset consists of around 250 ECG images of confirmed COVID-19 patients, 77 ECG images of Myocardial Infarction Patient, 203 patients of Previous History of Myocardial Infarction positive patients and total and 504 patients of abnormal heartbeats and finally 859 Normal Person ECG Images. The dataset which we use must contain only heart patients (our model diagnosis Covid-19 specifically for heart patients). So, the dataset must have two basic classes: ECG images of heart patient with positive Covid-19 case and ECG images of heart patient with negative Covid case. So, we updated the dataset, in this study [11], by reviewing the class of ECGs of Covid-19 cases with a cardiac diseases expert, and then dividing this class into two categories: heart patient with positive Covid-19 case and not-heart patient with positive Covid-19 case. Finally, we excluded the category of not-heart patient with positive Covid-19 case.

**Fig. 2:**
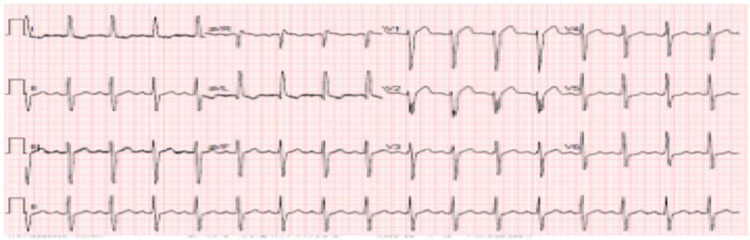
ECG of heart patient with positive Covid-19 case

Based on our update and the original dataset, we generate an updated dataset consists of 240 ECGs of heart patient with positive Covid-19 cases, as shown in Fig. 2, and 777 ECGs of heart patient with negative Covid-19 cases, as shown.

### Dataset pre-processing

Large dataset is a very important requirement to classify efficiently by deep learning models. However, large datasets are not always available as Covid-19 case, because it is a novel pandemic. So, the data augmentation techniques should be applied to increase the classification efficiency. Data augmentation technique has achieved the best results for deep learning models to handle the imbalance issue [30]. Some researchers tested specific augmentation techniques (flipping, cropping, perspective, contrast) on an ECG dataset. They found that these augmentation techniques reduce the accuracy of the model [31], because these techniques are not convenient to the nature of ECG (time series) instances. So, we decided to use zooming in and changing the brightness augmentation techniques because these techniques are more convenient to the nature of ECGs and do not affect the information in the ECG instances. Also, if we suppose that these techniques do not add very much information to the model, they still balance the classes and prevent the classifier to be biased toward the bigger class [32]. In this paper, changing the image brightness and zooming-in (randomly zooming the image by a certain range) augmentation techniques are used to increase the ECG images of Covid-19 patients. So, data augmentation presents data diversity and high accuracy for classification models.

Changing the image brightness and zooming-in augmentation techniques have been applied to ECGs which belong to the COVID-19 class, which has a limited number of samples. After applying the data augmentation process, the number of COVID-19 class images are raised, and the number of new COVID-19 class samples has become 580. The brightness of ECG images of the Covid-19 class are changed in a value from 0 to 5.0 according to a random generated number.

### The proposed model

As shown in Fig. 1, after data augmentation technique which we used above to balance the classes of the dataset, the transfer learning technique achieved the largest average accuracy for deep learning-based diagnostic models. Whereas the transfer learning technique helps the model by using CNNs that were trained before based on a huge dataset, and so these CNNs have an efficient experience in classifying and processing images. Also, ensemble learning has a great impact on improving the accuracy of the deep learning model by combining the advantages of more than one CNNs in one model to improve the accuracy. So, in our proposed model, we combine the transfer learning and the ensemble learning techniques to improve the accuracy and the performance of the model.

In this paper, 3 common pre-trained CNNs were utilized to classify COVID-19 cases from non-COVID-19 cases: (1) VGG-19, (2) AlexNet, (3) ResNet-101. Compared to VGG-16 model, VGG-19 is a deeper CNN architecture. AlexNet is a feedforward CNN with 8-layer depth [33].

There are two approaches to apply the ensemble technique: bagging and boosting approaches. We choose the bagging technique because it achieves more stability and accuracy compared to boosting approach [34]. Bagging approach decreases the overfitting issues, compared to Boosting approach, because, in each stage of the Boosting technique, the samples that are not classified correctly in the previous phase are only utilized as training data to the next CNN. In this paper, the Bagging technique trains all the 3 pre-trained models independently based on the same training set. Let n models numbered as 1, 2,…, n are used for classification of m classes, and the prediction probability values are denoted by P. The prediction probabilities for an image from model i can be described as a matrix as in Eq. 5.

$$ P_{1}^{\left( i \right)} = P_{1}^{(i)} P_{2}^{\left( i \right)} \ldots P_{n}^{(i)} $$

## Experimental evaluation

### Confusion matrix

Confusion matrix consists of four factors: True Positive (TP), False Positive (FP), True Negative (TN), and False Negative (FN), as shown in Table 2. In the confusion matrix, the rows contain the ‘Real class values’, and the columns are the ‘Predicted class values’. This evaluates the efficiency of our model. Based on the confusion matrix on the validation data set, our proposed model has achieved the sensitivity of 97.6% and specificity of 100%. Whereas Sensitivity points to the quantity of True Positives (TP) or the number of cases in which the model predicts they are Covid-19 patients and they actually Covid-19 patients. So, out of the 125 Covid-19 patients in the test data set we were accurately predicted COVID- 19 in 122 of them presenting 97.6% probability. This shows that we can diagnosis COVID-19 in the patients, with only 2.4% error. Specificity point to the quantity of True Negatives (TN) or the number of cases in which the model predicts they are not Covid-19 patients and they actually not Covid-19 patients. So, out of the 126 patients that are actually not infected by Covid-19, in the used validation data set, we accurately predict that these 126 persons are not infected by COVID-19 providing 100.00% probability. By calculating the accuracy of our model based on the confusion matrix we have achieved an overall accuracy of 99.1%.

**Table 2:** The confusion matrix of our proposed model

Predicted class		Covid	Other
Actual class	Covid	TP = 123	FN = 2
	Other	FP = 0	TN = 126

### Various evaluation metrics

**Fig. 3:**
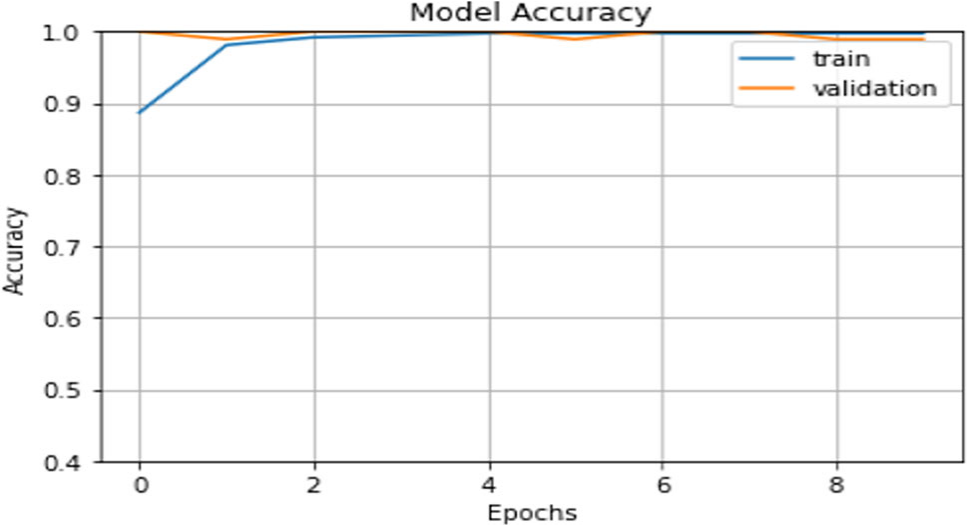
The training and validation accuracy of our proposed model

We have trained our proposed model for 10 epochs and the learning rate has been 0.0001. The validation accuracy, as shown in Figure 3, lies around 98.91–100%. The obtained results show that the validation accuracy could reach up to 100% which could be considered as one of the best Covid-19 diagnostic measures. As shown in Table 2, our approach practically outperforms [12–14, 35] models, which are the most high models until now, in accuracy, sensitivity and specificity.

### A novel comparison among the general Covid-19 diagnostic approach and our proposed approach

**Fig. 4:**
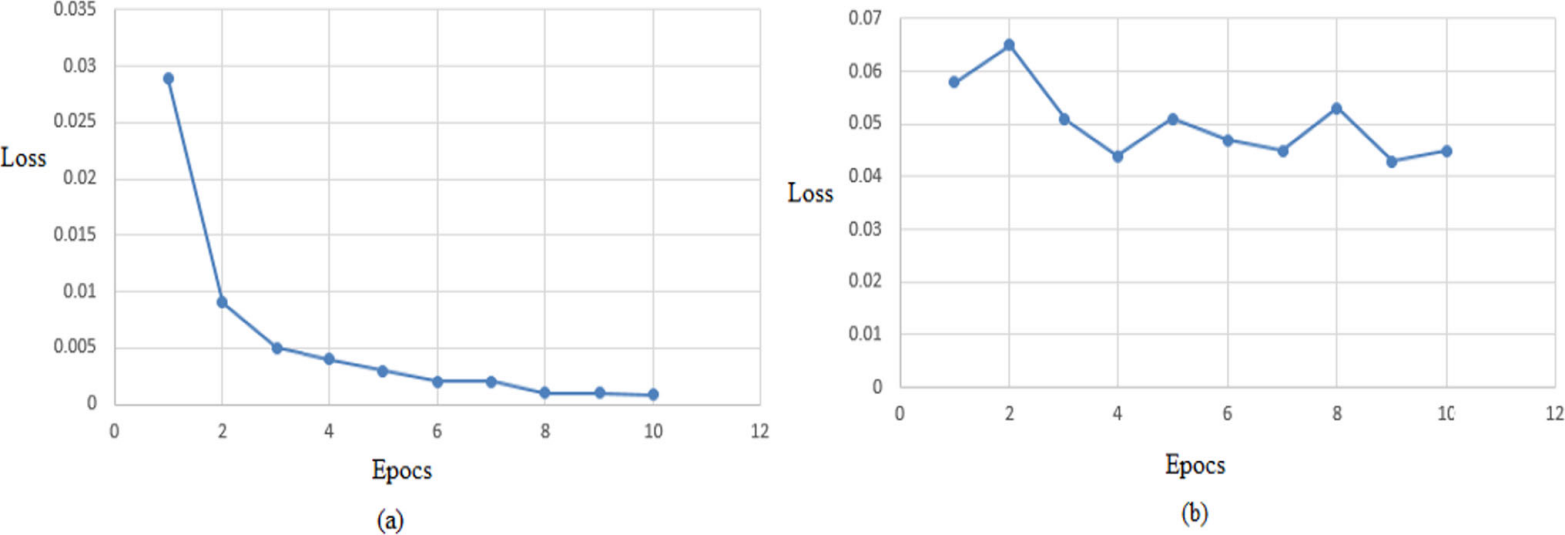
**a** Validation-loss of our proposed model vs **b** validation-loss of general Covid-19 diagnostic model

Compared to previous works that present general Covid-19 diagnostic models for any one regardless its health conditions, we present a Covid-19 diagnostic model for heart patients only. We compare the two approaches based on “validation-loss” metric as shown in Fig. 4. We have found that the validation loss metric in the case of general Covid-19 diagnostic model is unstable (transfers from up to down and vice versa). This indicates that there is dispersion in the data, so the learning process in not well. However, the validation- loss metric in our proposed approach (diagnostic model for only heart patients not any one) decreases in a continuous manner and reaches a very small value (0.0008) almost equal to zero. This indicates that our proposed model achieve better performance and learning.

### k-fold cross-validation

In order to evaluate the performance of the proposed methodology, a fivefold cross-validation is performed. The results of this evaluation metric is shown in Table 3. The average accuracy for all folds is 98.32%.

**Table 3:** Practical comparison among our approach and other general Covid-19 diagnostic models

Ref	Accuracy (%)	Sensitivity (%)	Specificity (%)
[13]	98.08	95.13	95.30
[14]	96.78	98.66	96.46
[35]	93.3	91	94
[12]	93	90	96
[27]	97.6	90.7	94.5
[29]	98.8	98.1	98.2
Proposed model	99.1	99	100

## Conclusion and future works

We have proved practically that using a general diagnostic model for Covid-19 is not efficient because in this case there is dispersion in the data which affects the performance. So, in this paper, we have proposed a deep learning-based Covid-19 diagnostic model for heart patients only. Our proposed model has achieved very efficient results. We handle the only one available dataset which contains ECGs for Covid-19 patients to be suitable to our model with the help of a heart diseases expert. We produce a new version of this dataset that consists of two classes: ECGs of heart patients with positive Covid-19 cases and ECGs of heart patients with negative Covid-19 cases.

In spite of our manuscript proposes an efficient and new approach for deep learning-based Covid-19 diagnostic model, our proposal stills having some pitfalls whereas the dataset is so limited that is not enough to achieve efficient generalization. Also, the deep learning technique is considered as a black box whereas it does not justify its predictions. So, we do not know why the model really outputs this classification, even if the result is accurate. In our future works, we intend to use the recent version of deep learning that is explainable deep learning that justifies the result of the model and clears the interested area of the model in the data samples that the model depends on them in its predictions. Therefore, we will try to handle all these drawbacks in our future works.

## Supplementary Information

Below is the link to the electronic supplementary material.

Supplementary file1 (PDF 18 KB)

## Data Availability

The original dataset is available at https://www.sciencedirect.com/science/article/pii/S2352340921000469, but we edited it to be convenient to our task. The edited dataset has not been online available yet.
