# Supplementary material for: A novel and accurate deep learning-based Covid-19 diagnostic model for heart patients
Source: Signal Image Video Process. 2023 May 19:1–8. Online ahead of print. doi: 10.1007/s11760-023-02561-8 (PMC10197036; doi:10.1007/s11760-023-02561-8)
Supplement: Supplementary file 1 — Supplementary file1 (PDF 18 KB) [file 11760_2023_2561_MOESM1_ESM.docx]

**Supplementary Table 2**: a comprehensive comparison among the most ten top cited papers of using deep learning in Covid-19 diagnosis

| Ref. | Dataset | | | | | | | Pre-processing | | | | Key points | Performance evaluation | | |
| --- | --- | --- | --- | --- | --- | --- | --- | --- | --- | --- | --- | --- | --- | --- | --- |
|  | type | Total | Covid-19  samples | bacterial pneumonia  samples | normal | Format | Dataset  publicly  available | Samples  exclusions | augmentation | Samples segmenta--tion | Re-sized |  | accuracy | sensitivity | Specificity |
| [11] | X-ray images | 1125 | 125 | 500 | 500 | PNG |  |  | _ | _ | _ | It develops a model using already pre-trained models such as Darknet-19. | 98.08% | 95.13% | 95.30% |
| [12] | X-ray images | 1428 | 224 | 700 | 504 | various |  | _ | _ | _ |  | The technique called Transfer Learning was applied. So, the detection of diverse abnormalities in small datasets is applicable. | 96.78% | 98.66% | 96.46% |
| [23] | X-ray images | 13962 | 358 | 5538 | 8,066 | various |  | _ | _ | _ | _ | According to the authors’ knowledge, this model is one of the first open source CNNs for the diagnosis of COVID‑19 from CXR images. | 93.3% | 91% | 94 |
| [10] | CT scans | 4352 | 1292 | 1735 | 1325 | N/A | - |  |  |  |  | For example, given a 3D CT scan, the paper first extract the lung region as the region of interest using a Unet (17) based segmentation technique. After that, this region of interest image is then transferred to COVNet for the classification. | 93% | 90 | 96 |
| [24] | CT scan | 274 | 15 | 259 | - |  |  |  |  |  |  | The architecture contains three basic processes: (1) preprocessing of the input images (selecting region of interest (ROI) from CT scan image) (2) training using ROIs and (3) classification and prediction of binary classifiers. | 89.5% | 87 | 88 |
| [25] | CT scan | 2006 | 1020 | 86 |  |  | - |  |  |  |  | Ten popular CNNs were used to classify the cases of COVID-19 and non-COVID-19 groups. | 91.51% | 93.8 | 89.9 |
| [26] | CT | - | - | - | - |  | - | - | | | | A convolutional neural network (CNN) is utilized to detect  COVID-19. Also, the initial parameters of the CNN are tuned by multi-objective differential evolution (MODE). | 93% | 90.7 | - |
| [27] | X-ray | 5310 | 76 | 4290 | 1583 |  |  |  |  |  |  | The network archicture is tuned for the COVID-19 detection with Bayesian optimization additive. Fine-tuned hyper parameters and also augmented dataset (to overcome the unbalance problem) make the proposed model achieves high efficiency. | 98.3% | 99.35% | 99.67% |
| [28] | ECGs | 1409 | 250 | 300 | 859 | N/A |  |  |  |  |  | a hybrid feature selection based on the chi-square test and sequential search to select the most important features. Also, it uses several machine learning classifiers to make two the classification process. | 98.2% | 90% | 95% |
| [30] | ECGs | 1937 | 250 | 300 | 859 |  |  |  |  |  |  | features are fused based on discrete wavelet transform (DWT) and integrated with fully connected features. After that, the size of the generated features is decreased in the feature selection stage. | 98.8% | 98.8% | 98.8% |
